# Genomic features, metabolism, and biotechnological applications of *Candida tropicalis* and other non-*albicans Candida* species

**DOI:** 10.1007/s11274-026-05245-w

**Published:** 2026-09-11

**Authors:** Thaynara Lorenzoni Entringer, Wendel Batista da Silveira

**Affiliations:** 1 Department of Microbiology, Universidade Federal de Viçosa, Av. P. H. Rolfs, s/n, Viçosa, MG 36570-900 Brazil; 2 Institute of Biotechnology Applied to Agriculture (BIOAGRO), Universidade Federal de Viçosa, Viçosa, MG 36570-900 Brazil

**Keywords:** Yeasts, Bioeconomy, Byproducts, Circular economy

## Abstract

The production of bio-based products by yeasts from agroindustrial byproducts is a key strategy for advancing circular bioeconomy. While *Saccharomyces* species remain the predominant industrial yeasts, their limited ability to assimilate lactose, pentoses, and glycerol, as well as their sensitivity to lignocellulose-derived inhibitors, restricts their efficient application in bioprocesses based on using industrial byproducts as fermentation media. In contrast, several non-*albicans Candida* species exhibit broad substrate utilization capacities and enhanced tolerance to industrial stresses, making them attractive candidates for the bioconversion of agroindustrial residues. This review critically examines recent advances in the genomic, metabolic, and physiological characterization of promising non-albicans *Candida* species, including *Candida tropicalis*, *Candida parapsilosis*, *Candida viswanathii*, *Candida sojae*, and *Candida maltosa*. Emphasis is given to genome-scale metabolic models, carbon assimilation pathways, stress-response mechanisms, and metabolic engineering approaches aiming at the production of value-added compounds. By identifying current achievements, knowledge gaps, and biotechnological bottlenecks, this review highlights the potential of these yeasts as emerging platforms for sustainable bioprocesses within a circular bioeconomy framework.

## Introduction

In a circular bioeconomy perspective, the use of agroindustrial byproducts such as lignocellulosic biomasses, crude glycerol and lactose-rich residues as substrates for microbial production of biobased products is of great interest to promote sustainable production chains, reduce greenhouse gases emissions, and avoid competition with food sources (Kumar et al. 2023). Although yeasts, mainly *Saccharomyces*, have been applied in bioprocesses for a long time, many industrial *Saccharomyces* strains have limited ability to use lactose and efficiently metabolize pentoses found in hemicellulosic hydrolysates or glycerol. In addition, these yeasts do not accumulate lipids and lipid derivatives, and have growth and productivity diminished in the presence of high concentration of inhibitors, mainly weak acids, furans, and phenolic compounds or at high temperatures (Cunha et al. 2019; Demeke et al. 2024).

Yeasts in the CTG clade, or CUG-Ser1 or Serinales, have attracted human attention for centuries due to their medical relevance. This group of ascomycetous yeasts was named after its unique ability to translate the CUG codon as serine (Ser) rather than leucine (Leu). Among *Candida* species, *C. albicans*, *C. tropicalis*, *C. dubliniensis*, and *C. auris* are clinically relevant (Pfaller et al. 2019). *Candida albicans* is a diploid, dimorphic yeast commonly found on the skin and mucosal surfaces as part of the normal human microbiome. However, it has considerable pathogenic potential and can cause infections under certain conditions, including weakened immunity (Parambath et al. 2024). Among the members of the CTG clade, there are also species and strains that are rarely isolated from humans with clinical relevance. These yeasts come from a variety of environments, including soil, air, water, and other animals, such as insects. Due to their phylogenetic proximity, CTG species without clinical associations offer a valuable point of comparison for better understanding the pathogenicity of these microorganisms. For instance, avirulence is not explained by the simple absence of pathogenicity genes, which are mostly conserved, but rather by regulatory and functional changes in these genes (Chávez-Tinoco et al. 2024). The view regarding pathogenicity has shifted from a binary perspective to a more continuous one. Environmental and clinical isolates of *C. parapsilosis* and *C. tropicalis* can share virulence-associated traits and genes. Considering organisms with an opportunistic potential, the disease onset depends on host context, route of exposure, and strain-specific traits, including host interaction capabilities, adhesion, biofilm formation, tolerance to stresses generated by host responses, and antifungal resistance (Abi-chacra et al. 2013; Jain et al. 2026; Pryszcz et al. 2013; Sabino et al. 2011; Zuza-Alves et al. 2017).

Thus, when considering applying *C. tropicalis* and *C. parapsilosis* in bioprocesses, some precautions and safety aspects should be considered. First, the yeast strain should be analyzed using genotyping tools, such as fingerprinting, multiplex PCR, microsatellite, or MALDI-TOF to verify its proximity to clinically associated strains. Then, the strains should be assessed in a phenotype panel with multiple parameters, including adhesion and biofilm formation capacity, presence of hydrolytic enzymes associated with host infection (e.g., lipases, proteases, hemolysin), tolerance to oxidative and nitrosative stress, ability to grow at 37 °C and antifungal susceptibility. Additionally, whole-genome sequencing can be used to track concerning genotypes and assess the copy number of genes related to resistance and outbreaks (e.g., EGR11, TAC1 variants) (Ryan et al. 2025; Zhang et al. 2026). When necessary, that is, if the presence of multiple virulence-associated traits is detected, in vitro screenings should be supplemented with host models or tissue/cell cultures. Ideally, strains for biotechnological applications should not be related to outbreaks, be drug-susceptible, have low-biofilm formation capacity, do not grow well on body temperature (close to 37 °C) and do not adhere to epithelial surface (De Melo Pereira et al. 2022; Kieliszek et al. 2017).

**Fig. 1:**
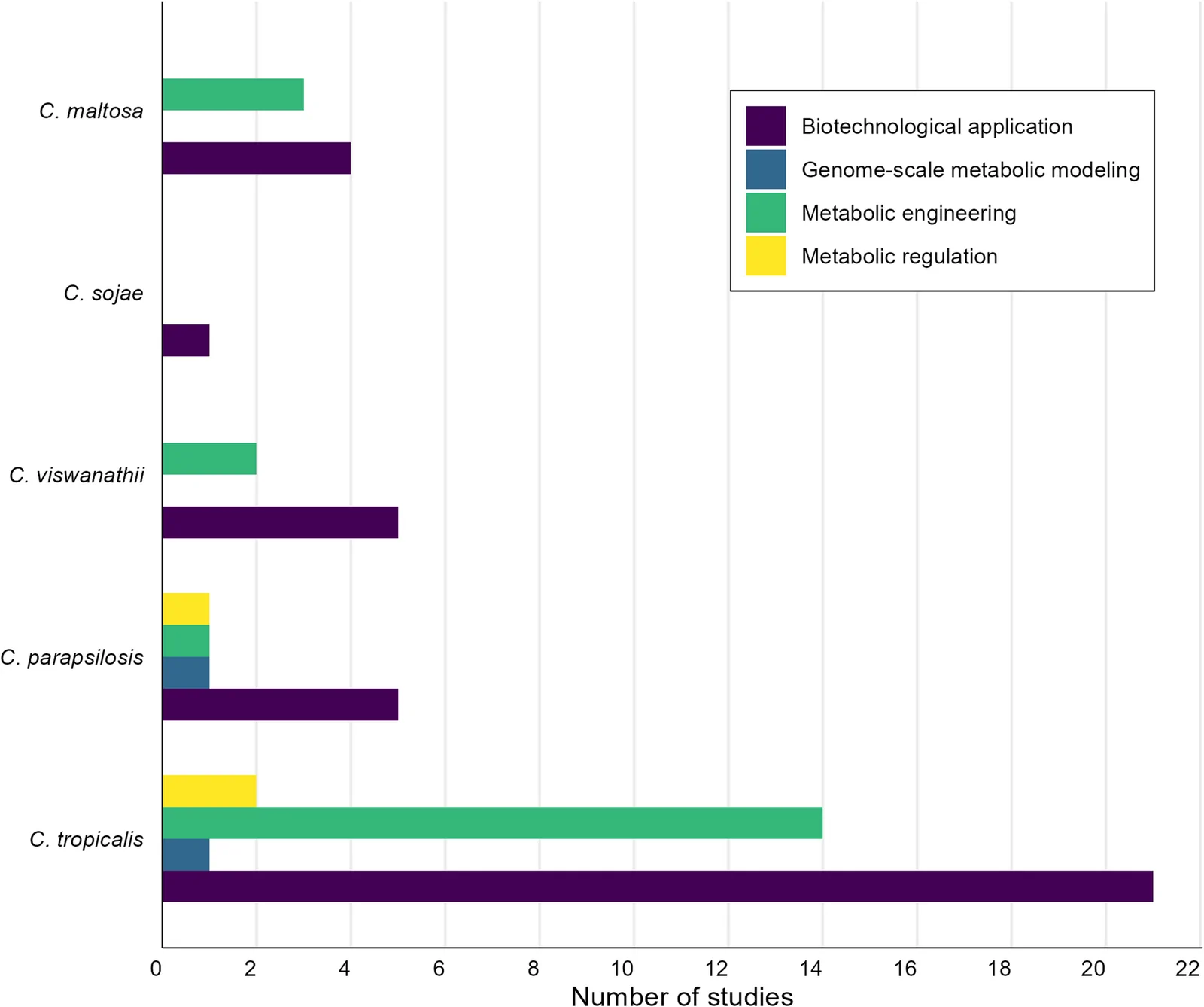
Number of studies published over the past 10 years (from January 2006 to March 2026) involving non-*albicans Candida* species (*C. tropicalis*,* C. parapsilosis*,* C. viswanathii*,* C. sojae* and *C. maltosa*) across four research categories: metabolic regulation, genome-scale metabolic models, biotechnological applications and metabolic engineering. Searches were conducted in PubMed and Google Scholar databases set the date from 2006 using the following terms: Candida; Candida AND metabolism; Candida AND metabolic regulation Candida AND biotechnology; Candida AND biotech; Candida AND biotechnological applications; Candida AND genome; Candida AND metabolic engineering; Candida AND GEM; Candida AND genome-scale metabolic model; Candida AND metabolic modeling; Candida AND metabolic model. Similar searches were conducted for each species considered here. Only peer reviewed manuscripts were included. Studies related to *Candida albicans* and duplicates were excluded, and only non-duplicated manuscripts were included. Only peer reviewed manuscripts were included. The categories presented were considered as follows: (i) biotechnological application: manuscripts that comprised only the application on bioprocessing, without developing novel strains; (ii) genome-scale metabolic modeling: reconstruction of curated metabolic models; (iii) metabolic engineering: studies focused on developing novel strains using genetic engineering tools or untargeted methods (e.g., adaptive laboratory evolution, mutagenesis); (iv) metabolic regulation: manuscripts focused on studying the metabolic responses to different environmental conditions (e.g., carbon sources, stressful conditions, growth inhibitors)

Meanwhile, although some *C. tropicalis* and *C. parapsilosis* strains can have clinical relevance, there are environmental and industrial isolated strains that have not been implicated in human infections. Among them, different strains of *C. tropicalis* stand out in biotechnological applications and metabolic engineering strategies (Fig. 1; refer to the “Biotechnology applications and metabolic engineering” section). For example, some strains, such as SY005 and DC-H21D, have been used as chassis for metabolic engineering to produce lipid derivatives (Queiroz et al. 2023). *C. parapsilosis* also has strains without clinical implications, especially those isolated from natural or industrial environments. Beyond sharing some characteristics related to carbon source assimilation with *C. tropicalis*, it also has attracted attention due to its efficient β-oxidation of fatty acids, which can be applied for bioremediation of oil-contaminated areas and for biosurfactant production (Csutak et al. 2024). Furthermore, *C. viswanathii*, has been reported as a promising producer of both lipase and α,ω-dicarboxylic acids (Cao et al. 2017). *C. maltosa* was broadly applied in the former Soviet Union as a single cell protein (SCP) source (Mauersberger et al. 1996) and has also been evaluated for xylitol production in a lignocellulosic biorefinery setup (Jiang et al. 2016). Additionally, *C. sojae*, which is commonly reported as a xylose fermenting yeast, has been poorly explored (Pant et al. 2022).

**Fig. 2:**
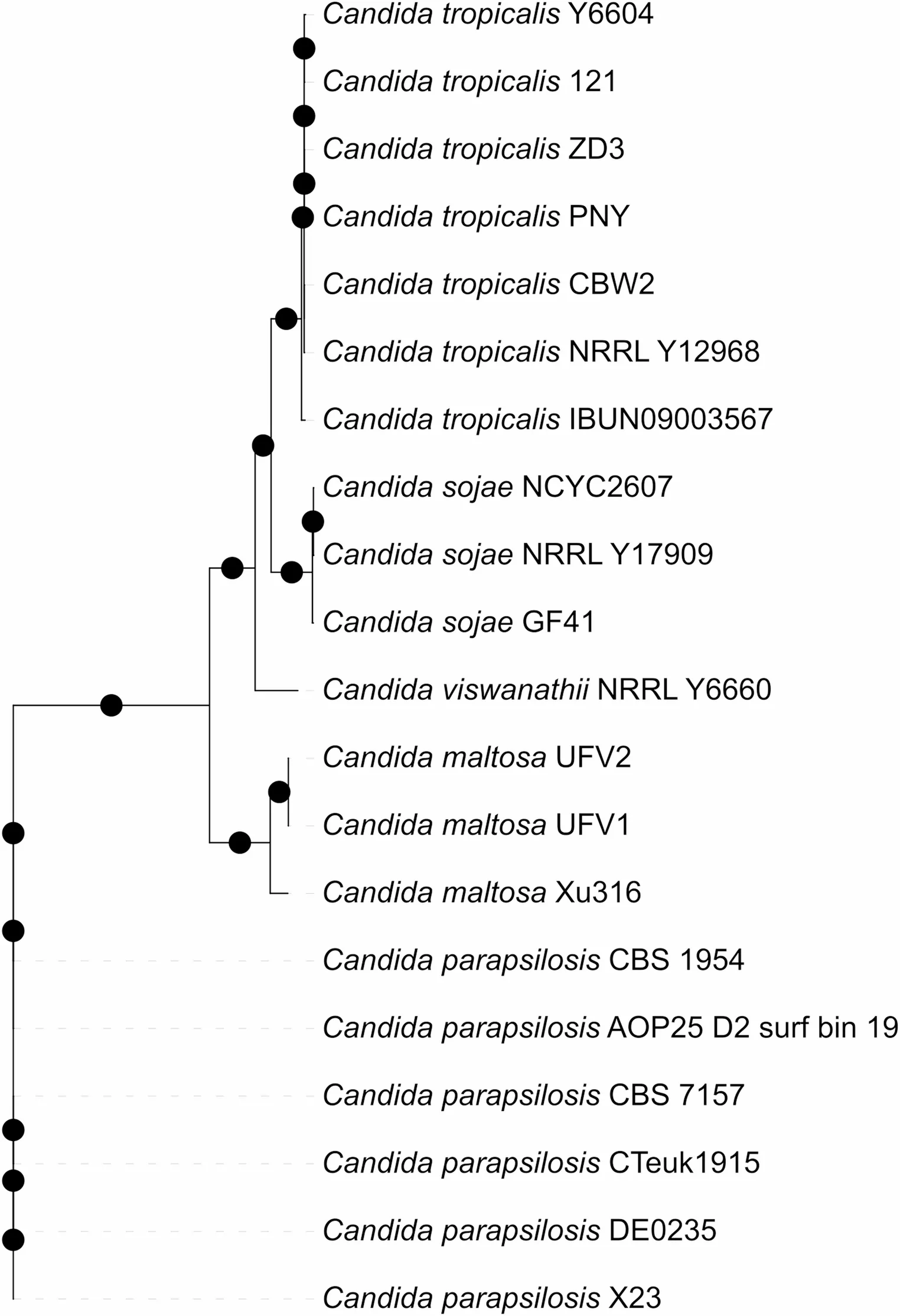
Phylogenomic tree of biotechnologically-related non-*albicans Candida* yeasts. The genomes were retrieved for environmental or industrial isolates in the GenBank database (O’Leary et al. 2024) and evaluated for quality (QUAST; Gurevich et al. 2013) and completeness (BUSCO; Tegenfeldt et al. 2025). After removing 4 genomes with low quality and completeness, 21 were selected for analysis and annotated in a standardized manner using the BRAKER 3 pipeline (Gabriel et al. 2024). Shared single copy protein orthologs between the genomes were identified using Orthofinder 3 (Emms et al. 2025) and aligned with MAFFT (Katoh 2002) with the MFP mode. The alignment was polished with trimAl (Capella-Gutiérrez et al. 2009) and the maximum likelihood tree was built using IQ-TREE 3 (Wong et al. 2025) with 1000 Ultrafast Bootstrap Approximations (UFBoot). In the tree, the black dots represent > 95% confidence nodes in the UFboot analysis

In this review, we present and summarize the current progress (last 10 years) and challenges regarding the biotechnological application and metabolic engineering of non*-albicans Candida* species, specifically *C. tropicalis*,* C. parapsilosis*,* C. viswanathii*,* C. sojae*, and *C. maltosa* (Figs. 1 and 2). It should be noted that some yeast species, previously belonging to the genus *Candida*, have been reclassified and are therefore not included in this review, specifically: *Candida glabrata* as *Nakaseomyces glabratus* (Opulente et al. 2024), *Candida glycerinogenes* as *Pichia kudriavzevii* (The Yeasts 2025), and *Candida famata* as *Debaryomyces hansenii* (The Yeasts 2025).

## Genomic features

Assembled genomes of non-*albicans Candida* yeasts typically range from 12 to 15 Mb. They are usually diploid, a characteristic shared with *C. albicans.* In contrast to *C. albicans*, which has 8 pairs of chromosomes, they have an average of 7 to 9 chromosome pairs (Odds et al. 2004; Chibana et al. 2005; Rustchenko 2007). In addition, some strains exhibit numerical variations due to aneuploidy, such as 19 or 21 chromosomes resulting from trisomy of chromosome 1 or 2 (Rustchenko 2007). The *C. maltosa* strain L4 frequently presents three chromosomes containing the *cm-ADE1* gene, although its genomic instability can result in variation of this number, with evidence of four or more chromosomes (Becher et al. 1995). *C. parapsilosis* exhibits chromosomal plasticity; while the reference genome for the species has 8 chromosome pairs (Mutalová et al. 2024), different isolates can have between 7 and 9 pairs (Fernando and Samaranayake 1998). *C. tropicalis*, in turn, has a complex chromosomal structure organized into seven pairs (Guin et al. 2020; Xu 2021).

It is worth noting that in GenBank, as of March 2026, 3 genomes are available for *C. maltosa*, 63 for *C. parapsilosis*, 58 for *C. tropicalis*, 3 for *C. sojae*, and 2 for *C. viswanathii*. The difference in the availability of sequenced genomes underscores that *C. parapsilosis* and *C. tropicalis* have been better characterized from a genomic point of view. Furthermore, to the best of our knowledge, there are no recent studies concerning the genome and genetic dynamics of yeasts from this genus related to biotechnological applications, such as changes in chromosome numbers under stress conditions. The reference genomes of these yeasts are shown in Table 1 and a phylogenomic tree built using genomes from industrial and environmental isolates is presented in Fig. 2.

**Table 1:** – Reference genomes available on GenBank database

Species and strain	Genome size (Mb)	Number of scaffolds	Access code (NCBI) or reference
*Candida albicans*	14.3	8	ASM18296v3
*Candida maltosa*	14	45	ASM5049571v1
*Candida parapsilosis*	12.9	363	ASM1534487v1
*Candida tropicalis*	14.6	23	ASM633v3
*Candida sojae*	15.1	40	ASM1317757v1
*Candida viswanathii*	14.3	337	ASM1534487v1

## Genome-scale metabolic models

Only three genome-scale metabolic models (GEMs) of *Candida* species have been reconstructed: *C. tropicalis*, *C. albicans* and *C. parapsilosis*. General features of these GEMs can be seen in Table 2. Since this review focuses on *Candida* species that display biotechnological potential, we underscore the GEM of *C. tropicalis.* As *Candida glabrata* has been reclassified as *Nakaseomyces glabratus*, the first GEM reconstructed for the genus *Candida* was of *C. tropicalis* (Mishra et al. 2016). The iCT646 model was developed using KEGG (Kanehisa et al. 2025) and BRENDA (Hauenstein et al. 2025) databases, followed by manual curation of elemental balances and assignment of cellular compartments with CELLO (Yu et al. 2006). The biomass equation incorporated experimental data on amino acid composition and literature data on lipids, with experimental validation of the elemental composition. The model was validated with chemostat data on glucose and other substrates, revealing a predominantly respiratory metabolism. The flux balance analysis demonstrated that *C. tropicalis* has a significantly higher turnover of fatty acids, acetyl-CoA, and cofactors compared to *S. cerevisiae*. In contrast to *S. cerevisiae*, the predictions indicated that the pyruvate pool generated by sugar catabolism is directed to fatty acid biosynthesis. Moreover, the flux variability analysis indicated that the pool of NADPH required for lipid accumulation was provided mostly by the pentose phosphate pathway. In a metabolic engineering context aiming at dicarboxylic acids (DCA) production, the simulations indicated that, in addition to interrupting β**-**oxidation, it is necessary to reduce the flux of fatty acids towards *de novo* synthesis to improve yield (Mishra et al. 2016).

**Table 2:** General characteristics of genome-scale metabolism models (GEMs) of *Candida* species

Species	Model	Reactions	Metabolites	Genes	Reference
*Candida tropicalis*	iCT646	1420	1147	646	Mishra et al. 2016
*Candida albicans*	iRV781	1221	927	781	Viana et al. 2020
*Candida parapsilosis*	iDC1003	1804	1278	1003	Viana et al. 2022

## Physiology and metabolism of *Candida* non-*albicans*

### Growth conditions and assimilation of carbon sources

In a circular bioeconomy perspective, the ability of yeasts to assimilate and ferment carbon sources derived from agroindustrial byproducts is essential, including pentoses and hexoses from lignocellulosic biomasses, lactose from dairy residues, and glycerol from biofuel production. Species of the genus Candida stand out due to their metabolic versatility; however, their capacity to utilize and ferment these carbon sources varies according to the species. In general, these yeasts can assimilate a wide range of carbon sources, while fermentation profiles and growth temperature ranges differ. A comparative summary of assimilated and non-assimilated carbon sources, fermentative capacity, and growth temperature ranges for some Candida species was constructed based on The Yeasts database (https://theyeasts.org/) (Table 3).

**Table 3:** – Physiological characteristics of *Candida* considering agroindustrial-derived carbon sources*

Yeast	Growth temperature range (°C)	Arabinose	Cellobiose	Ethanol	Galactose	Glucose	Glycerol	Lactose	Maltose	Rhamnose	Sucrose	Xylose
*C. tropicalis*	19 to 42	A+	A + F+	A+	A + F+	A + F+	A+	A + F-	A + F+	A+	A + F+	A + F-
*C. parapsilosis*	19 to 40	A+	A-F-	A+	A + F+	A + F+	A+	A-F-	A + F+	A+	A + F+	A + F-
*C. sojae*	19 to 37	A-	A+	A+	A + F+	A + F+	A+	A-F-	A + F-	A-	A + F+	A+
*C. viswanathii*	19 to 42	A-	A + F-	A+	A + F+	A + F+	A+	A-F-	A + F+	A-	A+	A + F-
*C. maltosa*	19 to 40	A-	A+	A+	A + F+	A + F+	A+	A- F-	A + F+	A-	A + F+	A + F-

A = assimilated (+) or not (-); F = fermented (+) or not (-). The absence of F or A represents that the information was not available

The analyzed genera exhibit a broad capacity to assimilate different carbon sources, alongside species-specific differences regarding fermentative performance and stress tolerance. Most species can assimilate the primary carbon sources derived from agroindustrial byproducts, including galactose, xylose, cellobiose and glycerol, although their ability to ferment these substrates is species-dependent. This indicates their suitability to be applied in biorefineries. Among the species analyzed, *C. tropicalis* displays the broadest substrate utilization profile and has been the most extensively explored in bioprocesses involving lignocellulosic biomasses. Conversely, *C. maltosa* and *C. viswanathii* stand out due to their ability to assimilate hydrophobic substrates, highlighting their potential for the valorization of hydrocarbon-rich byproducts. Recently, Rodriguez-Ocasio et al. (2022) assessed the capacity of *C. maltosa* to grow using monocarboxylic acids, dicarboxylic acids, fatty alcohols, alkenes, and esters as carbon sources in YNB minimal media. The yeast presented growth in myristic acid, 1-tetradecanol, n-tetradecane, n-octadecane, n-docosane, n-octadecene, n-docosene, and lauryl acetate, underscoring its capacity to grow on hydrophobic substrates, as well as its potential of application in bioprocesses based on hydrocarbon-rich agroindustrial byproducts.

### Metabolism: features and regulation

For instance, few studies have focused on understanding the regulation of carbon metabolism in non*-albican*s *Candida* species. Special attention has been given to the regulation of metabolism of hydrocarbons and aromatic compounds related to antifungal activity, which can also be the basis for further studies aiming at the degradation of those compounds in a bioremediation context.

Cillingova et al. (2022) demonstrated that *C. parapsilosis* has a sophisticated system for assimilating aromatic compounds. The assimilation is coordinated by the transcription factors Otf1p and Gtf1p, which specifically regulate the 3-oxoadipate (3-OAP) and gentisate (GP) pathways, respectively. The activation of these pathways induces an integrated cellular response, which includes the induction of β-oxidation, the glyoxylate cycle, peroxisome biogenesis, and an antioxidant mechanism. The 3-OAP and GP pathways, although presenting a similar transcriptional regulation, differ in their metabolic behavior; the 3-OAP pathway depends on the glyoxylate cycle, while the GP pathway does not. Li et al. (2020) evaluated the transport mechanism of n-hexadecane in *C. tropicalis*. The results demonstrated that the transport of n-hexadecane is an active, energy-dependent process. The proteomic analysis reinforced this conclusion, identifying the differential expression of membrane proteins involved in active transport pathways, such as endocytosis and phagocytosis, and in energy production processes. Considering the tolerance to hypersaline conditions and assimilation/degradation of azo dyes, Ren et al. (2026) isolated and characterized a novel strain of *C. tropicalis* (JH) from marine sediments and conducted an RNA-seq and enzymatic analysis in hypersaline medium containing the dye, or not. They showed that nicotinamide adenine dinucleotide dependent 2,6-dichlorophenol indophenol reductase, lignin peroxidase, manganese peroxidase and laccase were responsible for the degradation/assimilation of the dye and genes related to oxidoreductase activity, sugar transport, and halotolerance (e.g., potassium and sodium transport and synthesis of compatible solutes) were upregulated.

Many studies describe the ability of *Candida* species to grow in the presence of inhibitors, such as catechol, tyrosol, and phenol (Dias et al. 2021). However, few studies have evaluated the metabolic responses of non-albicans *Candida* metabolism to growth inhibitors in a biotechnological context. Wang et al. (2015) investigated the metabolic responses of *C. tropicalis* to furfural, phenol, and acetic acid during the bioconversion of xylose to xylitol. Metabolomic analysis revealed that the inhibitors caused significant changes in the metabolic profile. Twenty-five metabolites were identified as the most impacted, including amino acids (such as leucine, glycine, serine), organic acids from the citric acid cycle (succinate, malate, citrate), fatty acids (palmitic, octadecanoic), and sugars (trehalose). Furthermore, the NADH/NAD+ ratio was significantly higher in inhibitor-treated cells, indicating a disruption in cellular redox balance, which is directly linked to the efficiency of the bioconversion of xylose to xylitol. Recently, Freitas and Dias et al. (2026) isolated and characterized two novel strains of *C. maltosa* (UFV-1 and UFV-2) from agricultural soil. The two strains tolerated up to 2.0 g/L acetic acid, 0.5 g/L formic acid, 2.0 g/L furfural, and 4.0 g/L HMF in minimal media with xylose as the carbon source.

These studies reveal common features in the metabolic responses of non-*albicans Candida* species. First, carbon assimilation is strongly linked to broader cellular responses extending beyond substrate catabolism, including the activation of β-oxidation, the glyoxylate cycle, peroxisome biogenesis, redox balance, and antioxidant defenses. Another key point is that metabolic flexibility appears to be a hallmark of these yeasts, enabling the utilization of chemically diverse substrates such as hydrocarbons, aromatic compounds, and lignocellulose-derived molecules. Despite existing differences regarding the metabolism of these carbon sources, similar adaptive responses were observed, particularly the induction of transport systems, energy-generating pathways, and mechanisms protecting against oxidative stress. Furthermore, tolerance to environmental and process-related stresses seems to be associated with metabolic reprogramming aimed at maintaining cellular redox homeostasis. However, current knowledge remains fragmented and focused mainly on *C. tropicalis*. Comparative studies integrating transcriptomics, proteomics, metabolomics, and genome-scale metabolic models remain scarce, limiting a broader understanding of the regulatory networks underpinning substrate utilization and stress tolerance. Bridging these gaps will be useful for the rational engineering and industrial implementation of non-*albicans Candida* species.

## Biotechnology applications and metabolic engineering

Over the last ten years, most studies involving biotechnological applications and metabolic engineering of non*-albican*s *Candida* species have focused on *C. tropicalis*, with few studies directed toward other species. Table 4 presents a summary of studies with biotechnological applications focused on carbon sources and cultivation strategies, whilst Table 5 shows the metabolic engineering efforts for non-*albicans Candida* species.

### *Candida tropicalis*

#### Production of sugar alcohols

Tamburini et al. (2015) evaluated *C. tropicalis* DSM 7524 xylitol production under optimized conditions, that is, initial xylose concentration (80 g/L), pH (5.5), temperature (37 °C), and microaerophilic conditions (kLa = 2 h⁻¹). The scaling of the process to a 3 L fermenter employing a fed-batch strategy led to a higher yield, corresponding to 86.84% (w/w) of the maximum theoretical yield of 0.9 mol of xylitol per mol of xylose as defined by Barbosa et al. (1988). This* C. tropicalis* strain also presented a hyper-acidophilic behavior, characterized by the ability to grow and produce xylitol in pH as low as 1.5, which makes it particularly promising for industrial applications, as it allows operation under non-sterile conditions, reducing production costs. Moreover, Mateo et al. (2015) used olive tree pruning biomass as a raw material to produce ethanol and xylitol by *C. tropicalis* NBRC 0618. During the fermentative process, sequential production was observed: first ethanol (from glucose) and subsequently xylitol (from xylose). The overall yields ranged from 0.27 to 0.38 kg/kg for ethanol and from 0.12 to 0.23 kg/kg for xylitol, with the highest values achieved in hydrolysates obtained with hydrochloric acid pretreatment. Li et al. (2015) applied adaptive laboratory evolution (ALE) in corn cob hydrolysate to improve the xylitol production by *C. tropicalis* from an enzymatic hydrolysate of corn cob hemicellulose. The evolution and optimization of the production process led to a maximum xylitol yield of 75.14%.

Wang et al. (2016) established a strategy for producing xylitol from xylose mother liquor (xylose-rich waste from lignocellulosic biomass pretreatment) using a mixed culture of *C. tropicalis* X828 and *Bacillus subtilis*. In the microaerobic stage, the cells metabolized glucose and the inhibitors furfural and HMF, converting xylose to xylitol. Furthermore, Mattam et al. (2016) isolated and characterized a new strain of *Candida tropicalis *(MTCC 25057) with capacity for application in consolidated bioprocessing (CBP). The yeast expressed cellulases (exoglucanases and endoglucanases) and xylanases across a wide temperature range (32 and 42 °C) and in the presence of different lignocellulosic substrates. Under optimized conditions (cultivation at 42 °C in pretreated hydrolysate containing 0.5% wheat straw), the strain achieved significant enzymatic activities: 114.1 U/g of exoglucanase, 97.8 U/g of endoglucanase, and 689.3 U/g of xylanase. The same strain was able to ferment the sugars released from the biomass, aerobically converting 49 g/L of xylose into 15.8 g/L of xylitol and, subsequently, anaerobically fermenting 25.4 g/L of glucose to produce 7.3 g/L of ethanol. The strain also showed tolerance to common inhibitors in hydrolysates, such as furfural, HMF, and acetic acid. Additionally, Kumar et al. (2018) evaluated xylitol production from corn cobs. The most efficient conversion of xylan to xylose (70%) was achieved through hydrolysis with diluted nitric acid. Fermentation of the detoxified and neutralized hydrolysate by* C. tropicalis *MTCC 6192 resulted in a xylitol yield of 85%. The overall process allowed the conversion of 15% of the initial cob mass into xylitol. Xu et al. (2019) evaluated the production of xylitol from sugarcane bagasse hydrolysate using *C. tropicalis* 31,949. They optimized the fermentation conditions, determining the inoculum age (26 h), inoculum size (10%), and initial xylose concentration (100 g/L). Under optimized conditions, the strain was able to produce 62.98 g/L of xylitol.

From a screening of 218 non-*Saccharomyces* yeast strains, Morais Junior et al. (2019) selected and identified seven new *C. tropicalis* strains that grew in hydrolysate with a high acetic acid concentration (8.0 g/L) at pH 5.5. The most efficient strain, named *C. tropicalis* JA2, could produce xylitol with a yield of 0.47 g/g of consumed xylose. After optimization (pH, temperature, nutrient sources, and initial concentrations of xylose and inoculum), the JA2 strain achieved 109.5 g/L of xylitol, with a yield of 0.86 g/g of consumed xylose and a productivity of 2.81 g/L h. This performance was achieved in sugarcane bagasse hydrolysate containing 8.0 g/L of acetic acid and 177 g/L of xylose supplemented with nitrogen sources. Meanwhile, Zhang et al. (2021a, b) evaluated the conversion of xylose mother liquor into xylitol using engineered strains of *C. tropicalis*. First, the xylitol dehydrogenase gene was deleted to block the conversion of xylitol to xylulose, thereby accumulating the desired product. Next, an NADPH regeneration system was implemented through the heterologous expression of genes from *Yarrowia lipolytica* (6-phosphogluconate dehydrogenase and glucose-6-phosphate dehydrogenase), providing the necessary reducing cofactor for the efficient conversion of xylose to xylitol. After optimization of the fermentation process, the resulting engineered strain, *C. tropicalis* XZX-B4ZG, produced 97.10 g/L of xylitol from 300 g/L of XML in a 5 L reactor, achieving a productivity of 0.82 g/L h and a conversion rate of 92.40%.

Bevilaqua et al. (2023) proposed the co-production of xylitol and arabitol by *C. tropicalis* using sugarcane bagasse hemicellulosic hydrolysate. When the process was conducted in a bioreactor, the concentrations of 21.1 g/L of xylitol and 4.2 g/L of arabitol were achieved. The productivity and yields obtained were 0.24 g/L h and 0.74 g/g for xylitol, and 0.02 g/L h and 0.43 g/g for arabitol. Vardhan et al. (2023) evaluated the xylitol production by *C. tropicalis* from areca nut husk. Fermentation of the hydrolysate resulted in 5.88 g/L xylitol with 79.68% of yield. Bianchini et al. (2023) investigated rapid adaptation strategies (i.e., a small number of serial passages in the aimed condition to promote physiological adaptation) to improve the fermentative performance of *C. tropicalis* FT120037 for xylitol production from a combined hemicellulosic hydrolysate of sugarcane bagasse and straw. Among the tested strategies (pre-cultivation in original, concentrated, and concentrated/detoxified hydrolysates), pre-cultivation in the concentrated and detoxified hydrolysates were more effective. This condition increased the xylitol yield and the efficiency of xylose bioconversion to xylitol by 13.3%, the volumetric productivity by 7.1%, and the specific production rate by 9.7%. A 21.5% reduction in glycerol production was also observed, indicating a more inhibitor-tolerant phenotype. The combination of hydrolysate detoxification and yeast adaptation proved to be an effective strategy for mitigating toxicity and improving xylitol production. Additionally, Kaur et al. (2023) evaluated the potential for xylitol production from rice straw hemicellulosic hydrolysate using evolved strains of the yeast *Candida tropicalis*. ALE was conducted in rice straw hydrolysate, which led to a xylose assimilation increase of 76.42% in the best strain and a xylitol production increase of 7.54%.

Singh et al. (2023) used genetically modified strains of *C. tropicalis* to produce xylitol and ethanol with minimal waste generation from corn cobs. Through EMS mutagenesis and genetic engineering, strains with reduced xylitol dehydrogenase (XDH) activity were obtained, eliminating the need for a co-substrate for xylitol production. The heterozygous XYL2/xyl2Δ strain (*C. tropicalis* K21D) stood out, showing a 16% improvement in xylitol production from pure xylose compared to the parental strain. After culture medium optimization, this strain achieved a production of 132 g/L of xylitol. Using corn cob hydrolysate, the production was of 32.6 g/L of xylitol from the hemicellulosic fraction, 32.0 g/L of ethanol from the cellulosic fraction, and 13.0 g/L of Single Cell Protein (SCP) for animal feed. Furthermore, Sidana et al. (2024) investigated the use of lignocellulosic biomass from Arundo donax (giant reed) as a raw material to produce xylitol and ethanol by *C. tropicalis*. After detoxification of the hemicellulosic hydrolysate with activated charcoal, a xylitol yield of 0.54 g/g and a volumetric productivity of 0.274 g/L h were reached. In parallel, the cellulosic fraction underwent enzymatic hydrolysis, and the subsequent fermentation of the saccharified broth produced ethanol with a yield of 0.47 g/g and a productivity of 0.63 g/L h. Kumar et al. (2026) isolated a novel strain of *C. tropicalis* (S7) from banana residues. This strain could grow on potato dextrose broth supplemented with 6 g/L of acetic acid and efficiently assimilated xylose with a 0.81 g/g xylitol yield.

Queiroz et al. (2025) investigated the influence of the glucose/xylose ratio and pH on the fermentation and gene expression of the yeast *C. tropicalis* for xylitol production. The condition with 50 g/L of xylose and pH 3.5 was the most efficient, resulting in the production of 29.81 g/L of xylitol, with a volumetric productivity of 0.52 g/L h and a yield of 0.54 g/g in 48 h. Concurrently, the expression of the xylose transporter genes (XUT1 and STL2) and the key enzyme xylose reductase (XYL1) was evaluated. The upregulation of XUT1 was correlated with higher xylose concentrations, while STL2 was favored at lower concentrations. The expression of XYL1 increased over time with higher xylose proportions. Based on these results, the authors hypothesized that the Xut1p-mediated xylose transport occurs via a proton symport mechanism and that pH indirectly influences the transcription of the XUT1 gene. This work highlights the importance of modulating these factors to optimize xylitol production from hemicellulosic hydrolysates. Eraso et al. (2025) evaluated xylitol production from rice husks using* C. tropicalis.* A rapid yeast adaptation strategy was employed, with a gradual 20% increase in hydrolysate concentration in the medium every 48 h. The optimal conditions (4.41 g/L of inoculum and 68.28 g/L of xylose) allowed the adapted strain to produce 36.74 g/L of xylitol, with a yield of 0.58 g/g of xylose and a volumetric productivity of 0.34 g/L h.

Overall, these studies point out that *C. tropicalis* is the most well-established non-*albicans Candida* platform for xylitol production. Three main trends can be identified: first, high xylitol yields were recorded from cultivations in lignocellulosic hydrolysates derived from agricultural byproducts such as sugarcane bagasse, corncobs, rice husks, and banana pseudostems, Second, tolerance to inhibitors is a decisive factor for process performance; consequently, detoxification procedures, adaptation strategies, and strain selection often lead to improved productivity. Lastly, recent metabolic engineering efforts have focused on cofactor regeneration, transport regulation, and metabolic pathway optimization, enabling to achieve xylitol tiers superior to 100 g/L. Taken together, these findings indicate that the primary bottleneck is no longer substrate utilization itself, but rather the optimization of carbon flux and inhibitor resistance under industrially relevant conditions.

#### Production of biofuels

Nouri et al. (2017) isolated and characterized a new thermotolerant strain of *C. tropicalis* (HNMA-1) with the ability to hydrolyze xylan for bioethanol production from sugarcane bagasse hydrolysate. The strain maintained significant xylanase activity at 40 °C (0.211 IU/mL) and exhibited growth at this higher temperature in both xylose medium and hydrolysate. However, ethanol production efficiency was affected by the temperature, with lower yields at 40 °C compared to 30 °C. Mahakuntha et al. (2021) evaluated the kinetics of both ethanol and biomass production by *C. tropicalis* TISTR 5306. This strain was cultivated in media containing longan juice and solid longan waste extract, in batch and fed-batch modes. In batch cultivations, longan juice presented the best results, with an ethanol concentration of 25.5 ± 0.8 g/L, an ethanol yield of 0.491 ± 0.017 g/g, and the highest specific activity of the enzyme pyruvate decarboxylase (PDC). This medium was selected for pulsed fed-batch cultivation, which resulted in an increase in ethanol concentration to 28.3 ± 0.5 g/L after the second feeding (120 h). The maximum PDC activity (0.057 U/mg protein) was observed during the first feeding phase.

Meanwhile, Thangavelu et al. (2020) developed a strategy for bio-oil production from cassava processing industry wastewater using *C. tropicalis* ASY2. The selected strain demonstrated the ability to use starch from the effluent (4–7%) to accumulate lipids, achieving a productivity of 0.010 g/L h and a lipid content of 49% (w/w). The fatty acid profile comprised a high oleic acid content (41.33%), a favorable characteristic to be used as feedstock for biodiesel production. Chattopadhyay and Maiti (2020) evaluated the potential of different isolates for biomass and lipid production in a medium containing a mixture of glucose and xylose. *C. tropicalis* SY005 demonstrated the highest lipid productivity (0.792 g/L d). Chattopadhyay et al. (2020) also developed a specific, marker-based, integrative genetic transformation system for *C. tropicalis* SY005. They ectopically expressed the strain’s own transcription factor CtRRAP1 (repressor activator protein 1), which resulted in a substantial increase in lipid accumulation, with the engineered strain reaching a maximum lipid content of 37% (w/w) after induction with galactose. This value represents a 60% increase compared to the wild-type SY005 strain.

Luo et al. (2023) studied a new strategy to obtain thermotolerant and efficient yeast strains for biodiesel production from lignocellulosic biomass. They employed a combination of ALE and room-temperature plasma mutagenesis to obtain a *C. tropic*alis strain capable of growing at 45 °C. They then performed two rounds of fluorescence-activated cell sorting (FACS) to isolate cells with high lipid content, resulting in a 16.8% increase in lipid accumulation capacity compared to the parental strains. The final strain was used in a simultaneous saccharification and fermentation (SSF) process with pretreated corn straw at 44 °C, achieving a lipid yield of 86.2 ± 2 mg/g of biomass.

#### Production of dicarboxylic acids (DCAs)

The ability of *C. tropicalis* to produce dicarboxylic acids has also been studied. Wang et al. (2018) proposed markerless genomic editing for *C. tropicalis*, using the *E. coli maxF* gene as a counter-selection marker on a suicide plasmid (pPICPJ-maxF). Initially, the carnitine acetyltransferase (CART) gene was deleted to reduce the β-oxidation of fatty acids, increasing DCA production from 4.14 to 8.27 g/L. Two homologous recombination steps were performed to replace the native promoters of the cytochrome P450 gene and the NADPH–cytochrome P450 reductase gene with the constitutive pGAP promoter. The resulting strain, *C. tropicalis* PIPP1702, achieved 11.39 g/L DCA in flasks. Finally, in a fed-batch fermentation process, DCA production was increased to 32.84 g/L (11.4-fold increase compared to the original strain).

Ju et al. (2020) proposed a strategy to produce adipic acid, an important monomer for the nylon industry, through metabolic engineering of *C. tropicalis*. They constructed strains deficient in acyl-CoA oxidase (*AOX)* genes, key enzymes in the β-oxidation pathway that degrade the desired intermediates, which increased adipic acid production from methyl laurate. The most efficient strain, *C. tropicalis* Δ*AOX*4:*AOX*5, produced 339.8 mg/L in flasks (5.4-fold increase compared to the parental strain). When subjected to a batch fermentation process with optimized conditions (agitation at 1000 rpm, pH 5.0, and 3% methyl laurate), this strain reached 12.1 g/L adipic acid, with a productivity of 0.1 g/L h. Moreover, the production of DCAs from alkenes or fatty acids relies on transporters located in the cellular and peroxisomal membranes. Zhang et al. (2021a, b) identified two putative proteins involved in this transport, CtFat1p (cell membrane) and CtPxa1p (peroxisomal membrane), and investigated their effects on DCA synthesis using homologous recombination to knockout the corresponding genes. Knocking out of *ctfat1* and *ctpxa1* genes drastically reduced DCA yield (56.19 and 88.38%, respectively). In contrast, knocking out a single copy of the *ctpxa1* gene increased the yield by 21.90%, suggesting that this modification may reduce excessive oxidation of fatty acids in peroxisomes, preferentially directing them towards DCA production. Finally, by increasing the *ctfat1* copy number, the intracellular fatty acid transport rate increased, as well as DCA production by 30.10%.

Gilis et al. (2026) assessed the potential of grease trap waste as a sustainable substrate for DCA production. First, the authors optimized the glucose and grease waste feed rate on fed-batch cultivations, as well as the pH, and reached a titer of about 35 g/L of DCA. They showed that low glucose feed rates (below 0.38 g/L h) were insufficient to sustain growth and maintenance, and higher feed rates (above 0.38 g/L h) induced excessive biomass formation. These results indicate the importance of fine-tuning cultivation conditions, besides strain improvements, for sustainable DCA production.

Taken together, these studies highlight key trends regarding the use of *C. tropicalis* for biofuel and lipid production. As pointed out in the previous section, *C. tropicalis* exhibits great substrate flexibility, capable of utilizing a wide range of agroindustrial feedstocks. Lipid accumulation is strongly linked to efficient xylose utilization and can be significantly enhanced through strain improvement strategies, including genetic engineering and ALE. The overexpression of regulatory factors, such as CtRRAP1, as well as the combination of ALE, mutagenesis, and cell screening approaches, have consistently resulted in increased lipid accumulation and improved process performance. Overall, the main challenges for industrial implementation lie not in substrate utilization itself, but rather in increasing lipid productivity, thermotolerance, and process robustness under large-scale cultivation conditions.

#### Production of other metabolites

The biotechnological versatility of *C. tropicalis* is also evidenced by its capacity to produce metabolites of industrial interest. α-Humulene is a plant-derived sesquiterpene with pharmacological activity, but the direct extraction from plants is unsustainable and low-yielding. To overcome this limitation, Zhang et al. (2022) developed a production process using *C. tropicalis* as a host for the *de novo* synthesis of α-humulene from glucose. They co-expressed an optimized α-humulene synthase gene with the endogenous farnesyl diphosphate (FPP) biosynthetic pathway. To further increase production, metabolic bottlenecks were overcome by overexpressing the rate-limiting enzymes α-humulene synthase, acetyl-CoA thiolase, and NADH-dependent HMG-CoA reductase. Combined with medium optimization, the best *C. tropicalis* strain produced 195.31 mg/L of α-humulene in shake flasks and 4,115 mg/L in a bioreactor via fed-batch, representing 253 and 5,345-fold increases compared to the initial production, respectively.

Li et al. (2022) proposed a versatile tRNA-based guide RNA (gRNA) platform in *C. tropicalis* for β-carotene production. The platform uses a specific tRNA (tRNA-Gly) from *C. tropicalis* to leverage the endogenous processing system and produce multiple mature gRNAs from a single transcript. By coupling the tRNA: gRNA platform with a dCas9, they developed the first CRISPR interference (CRISPRI) system for *C. tropicalis*. This system could silence exogenous (GFP) and endogenous (*ADE2* and *ERG9*) genes, reducing transcript levels by up to 23.9%. The practical application was demonstrated by silencing *ERG9* to redirect metabolic flux and increase β-carotene biosynthesis. Zhang et al. (2024) redesigned the biosynthetic pathway for the diterpene cembratriene-ol (CBT-ol), a potential biopesticide, and developed an engineered strain of *C. tropicalis* that achieved 1,425.76 mg/L CBT-ol in a bioreactor. The engineering strategies included compartmentalization of enzymes in peroxisomes, truncation of the key enzyme CBT-ol synthase, and overexpression of rate-limiting enzymes.

Li et al. (2024) proposed the metabolic engineering of *C. tropicalis* for the efficient production of 1,2,4-butanetriol, a precursor for pharmaceuticals and energetic plasticizers. To overcome the accumulation of xylonate due to low activity of heterologous xylonate dehydratases, they modulated iron metabolism, increasing the availability of intracellular ferrous ions and raising enzymatic activity by 2.2-fold. They also overexpressed key genes in the cofactor regeneration pathway, reaching 3.2 g/L 1,2,4-butanetriol. Moreover, the introduction of calcium carbonate to maintain pH balance further increased the titer to 4 g/L, representing 111% improvement over the base strain. Lastly, using corn cob hydrolysate as a substrate resulted in 3.42 g/L 1,2,4-butanetriol. Yang et al. (2026) recently developed strains of *C. tropicalis* capable of producing (−)-α-bisabolol, a sesquiterpene with anti-inflammatory and bactericidal properties naturally produced by chamomile. They co-expressed the MrBBS and ERG20 genes using a novel Linker- and Scaffold-Mediated Assembly Regulation Tool, combined with multiple knockouts (DPP1, Cat2, ERG10, ADH1, GPD1) and controlled regulation of enzymes in the acetyl-CoA/mevalonate pathway. First, the authors demonstrated the construction of viable (−)-α-bisabolol-producing strains, reaching titers between 260 and 291 mg/L. After optimization, a titer of 36.9 g/L was achieved on a 5 L fed-batch system.

These studies demonstrate a clear transition in the biotechnological application of *C. tropicalis*, shifting from traditional fermentation products to the synthesis of high-value specialty chemicals. A common feature of these processes is the extensive use of metabolic engineering to redirect carbon flux, increase precursor availability, and optimize cofactor regeneration. Strategies such as the overexpression of rate-limiting enzymes, pathway compartmentalization, transcriptional regulation, and CRISPR-based tools have enabled substantial improvements in product titers. The production of terpenoids stands out as one of the most promising applications, with engineered strains achieving concentrations in the range of grams per liter for compounds such as α-humulene, cembratrien-ol, and (−)-α-bisabolol. Concurrently, the development of genetic tools (including CRISPR interference systems and multiplex gRNA platforms) has expanded the capacity for rational strain engineering in this species. These advances position *C. tropicalis* as a versatile chassis for industrial biotechnology, although further improvements in pathway balancing, precursor supply, and process scalability are still required to enable commercial implementation.

### *Candida parapsilosis*

#### Production of sugar alcohols

Current evidence indicates that *Candida parapsilosis* is a promising platform to produce sugar alcohol, particularly mannitol and arabitol. Unlike *C. tropicalis*, which has been extensively explored for xylitol production, studies involving *C. parapsilosis* have focused primarily on converting glucose and arabinose into polyols with potential applications in the food and pharmaceutical industries.

A recurring trend across these studies is the strong influence of cultivation strategy and strain improvement on product accumulation. High sugar concentrations and fed-batch operation have proven effective approaches for increasing mannitol titers and preventing product reutilization. Meng et al. (2017) isolated the strain *C. parapsilosis* SK26.001 from sugarcane juice and demonstrated its potential for mannitol production from high glucose concentrations. Mannitol production reached 68.5 g/L in shake flasks and 80.3 g/L in benchtop bioreactors after 72 h; however, the produced mannitol was consumed once glucose was depleted. The fed-batch fermentation prevented mannitol reutilization and resulted in 97.1 g/L after 120 h. Similarly, Kordowska-Wiater et al. (2017) isolated and identified the yeast *Candida parapsilosis* 27RL-4 as an efficient arabitol producer. The 27RL-4 strain reached 10.42–10.72 g/L arabitol from 20 g/L of L-arabinose, with a yield of 0.51–0.53 g/g under optimized conditions. Arabitol production was favored under optimized nutritional conditions, including reduced nitrogen availability and controlled pH, suggesting that metabolic flux toward polyol synthesis is highly dependent on cultivation conditions.

Although natural isolates already demonstrate considerable capacity for polyol production, genome shuffling and mutagenesis strategies have resulted in significant gains in arabitol yields, highlighting the potential of non-targeted genetic improvement approaches for this species. For example, Kordowska-Wiater et al. (2018) used this strategy to generate more efficient strains of *C. parapsilosis*. The best strains, GSII-3 and GSII-16, showed a 50% increase in productivity compared to the original one and, under optimized cultivation conditions, reached a titer of 11.8 g/L (15–16% more efficient).

Taken together, these studies indicate that *C. parapsilosis* possesses a robust physiological capacity for sugar alcohol production, although research remains limited compared to *C. tropicalis*. Future efforts should focus on expanding the range of substrates evaluated, developing advanced genetic engineering tools, and assessing the performance of modified strains using agro-industrial raw materials. 

#### Production of other metabolites

In addition to sugar salcohol production, *C. parapsilosis* also displays the capacity toynthesize a wide range of metabolites with applications in the pharmaceutical, environmental, and chemical industries. A common feature in these studies is the ability to utilize low-cost or unconventional substrates, including sugarcane molasses, hydrocarbon-contaminated environments, and industrial waste. El-Gamal et al. (2018) evaluated citric acid production by *C. parapsilosis* NH-3 from sugarcane molasses. The optimal conditions for maximum production were 40 °C, pH 5.0, a concentration of 30% molasses pretreated with EDTA, nitrogen and phosphorus limitation, and the addition of 1% methanol, reaching a titer of 4.30 g/L after 24 h.

Furthermore, Garg et al. (2018) isolated a strain of *C. parapsilosis* capable of producing a biosurfactant from contaminated dairy products. The biosurfactant was identified as 13-docosenamide, which proved to be a broad-spectrum antibacterial agent, inhibiting the growth of *E. coli* and *S. aureus.* Csutak et al. (2024) assessed the biosurfactant production potential of *C. parapsilosis* CMGB-YT, isolated from oil-polluted soil. The strain could assimilate hydrocarbons, such as n-hexadecane, and produce an anionic glycolipid biosurfactant. The biosurfactant improved the growth of *Rhodotorula mucilaginosa* in wastewater contaminated with heavy metals (lead and cadmium), increased Chemical Oxygen Demand (COD) removal by up to 80%, and enhanced Cd²⁺ removal by 10%. 

### *Candida viswanathii*

#### Production of lipids

*C. viswanathii* has also been considered a promising oleaginous yeast due to its broad substrate versatility and its ability to accumulate lipids from agro-industrial feedstocks. Ayadi et al. (2016) showed that strain Y-E4 efficiently assimilates C5 and C6 sugars, glycerol, and hydrophobic substrates; notably, fed-batch cultivation substantially increased lipid accumulation to about 50% (w/w). Regardless of the carbon source, oleic acid remained the predominant fatty acid, indicating a relatively stable lipid profile under varying cultivation conditions. Guerfali et al. (2020) demonstrated that crude glycerol from biodiesel production can be efficiently converted into lipids by the same strain. Process optimization increased lipid accumulation to 52% (w/w), with an increase in polyunsaturated fatty acids, particularly linoleic acid, thereby confirming the strain’s potential for application in biodiesel production. Taken together, these studies highlight the potential of *C. viswanathii* as a sustainable microbial platform for lipid production.

#### Production of dodecanedioic acid (DDA)

Studies demonstrate that high DDA titers depend on the integration of suitable substrates, metabolic engineering, and process optimization. Cao et al. (2017) showed that strain the IPE-1 efficiently co-assimilates sugars (glucose and xylose) derived from lignocellulosic biomass and n-dodecane, resulting in an approximately 40% increase in DDA production (titer of 128.7 g/L) compared to cultivation using only glucose. This finding underscored the potential of combining renewable carbohydrate streams with hydrophobic substrates to enhance process efficiency.

Recently, the focus of research has shifted toward genetic engineering strategies. Pham et al. (2023) established a CRISPR/Cas9 platform to optimize the ω-oxidation pathway and demonstrated that the coordinated overexpression of genes involved in hydrocarbon oxidation and cofactor regeneration [*CYP52A19* (cytochrome P450), *CPRb* (cytochrome P450 reductase), *FAO2* (fatty alcohol oxidase), and *POS5* (ATP-NAD(H) kinase)] increased DDA biosynthesis while simultaneously reducing byproduct accumulation. The final strain doubled DDA production, achieving a titer of 224 g/L, a molar conversion of 83%, and a productivity of 1.87 g/L h. Next, Pham et al. (2026a) demonstrated that replacing expensive culture medium components with low-cost alternatives and optimizing feeding strategies significantly reduced production costs while maintaining high DDA titers. They evaluated different supplements to replace YNB medium and a combination of soy peptone, trace elements and vitamins reduced the medium cost by about 29 times. Then, they optimized surfactant addition, nutrient feeding rate (fed-batch cultivations) and cell density at the beginning of the DDA production phase and reached a DDA titer of 90–162 g/L. Further, Pham et al. (2026b) developed CRISPR activation (CRISPRa) system based on dCasMINI, enabling the programmable activation of endogenous genes. Among the tested activation domains, fusion with Med2 (dCasMINI-Med2) yielded the highest levels of gene activation with minimal impact on cell growth. To improve DDA production, a dodecane-inducible promoter was employed, reducing metabolic burden and increasing production by 11.6%. Additionally, activation of transporter genes improved DDA titers, with STL1_4 and HST6 leading to increases of up to 16%. These results illustrate how gene expression regulation can complement metabolic pathway engineering. Overall, these studies demonstrate that various strategies can be employed to improve production titers, and that combining techniques can yield even more promising results. Furthermore, *C. viswanathii* stands out as one of the most important non-*albicans Candida* species for the industrial production of dicarboxylic acids, although further research is needed regarding large-scale cultivation and the expanded use of low-cost, renewable raw materials to make commercial implementation viable.

#### Production of enzymes

Gomes et al. (2018) demonstrated that the yeast *C. viswanathii *can produce high levels of lipase in submerged culture and that Amazonian vegetable oils can successfully replace olive oil as carbon sources. Among the oils evaluated, pequi oil was the most effective inducer of lipase production (1.66 U/mL), while organic nitrogen sources consistently outperformed inorganic ones. These results suggest that renewable regional raw materials can enable efficient enzyme production while reducing reliance on conventional substrates.

### *Candida sojae*

Although *C. sojae* is still less explored than other non-*albicans Candida* species, it displays potential for lignocellulosic bioconversion. Pant et al. (2022) demonstrated that *C. sojae* JCM 1644 efficiently converted *Brassica juncea* hydrolysate into both ethanol and xylitol in a two-stage fermentation process, achieving yields of 0.45 and 0.62 g/g, respectively. The superior performance compared to single-stage fermentation (ethanol and xylitol yields were 15.57% and 11.78% higher in the two-stage process) reinforces that the metabolic flexibility of *C. sojae* can be effectively harnessed through appropriate process design (Pant et al. 2022).

### *Candida maltosa*

*C. maltosa* can assimilate diverse carbon sources, tolerate inhibitors, and produce industrially relevant compounds (Brown et al. 2023; Freitas and Dias et al. 2026; Jiang et al. 2016; Noroozi et al. 2026; Rodriguez-Ocasio et al. 2026). Over the last years, genetic engineering tools have been developed. Therefore, *C. maltosa* is a promising yeast to be applied in biorefineries.

In this sense, the ability of *C. maltosa* to efficiently metabolize pentoses has been successfully exploited for xylitol production from lignocellulosic feedstocks. Jiang et al. (2016) developed *C. maltosa* strains for xylitol production from corn cob hemicellulosic hydrolysate through ALE. The evolution process was conducted in sequential batches containing the corn cob hydrolysate supplemented with peptone and yeast extract. The best evolved strain achieved high productivity, yield, and xylitol concentration (> 100 g/L in 48 h), making it a promising and economically viable alternative. Rather than relying on genetic engineering, this study showed that evolutionary approaches can enhance the physiological robustness of *C. maltosa* under industrially relevant conditions.

Beyond carbohydrate-based substrates, *C. maltosa* has proven capable of utilizing hydrophobic carbon sources, expanding its potential application to areas such as bioremediation. Brown et al. (2023) demonstrated the species’ ability to grow on monomers derived from the thermal oxidative degradation of polyethylene (TOD_HDPE). Subsequent studies focused on overcoming the nutritional and physicochemical limitations associated with these poorly soluble substrates. Noroozi et al. (2026) showed that organic nitrogen sources, particularly algae extract and casein amino acids, increased biomass formation on both model hydrophobic compounds and TOD_HDPE. Based on these findings, Rodriguez-Ocasio et al. (2026) employed ALE to further enhance substrate assimilation, obtaining evolved strains with a specific growth rate twice that of the parental strain. It is important to highlight that the improved phenotype was associated with physiological adaptations, including increased biosurfactant secretion and lower membrane ergosterol content, suggesting that modifications affecting substrate accessibility and membrane permeability are determinants for the efficient utilization of hydrophobic substrates.

Freitas and Dias et al. (2026) showed that *C. maltosa* can be used to produce lipids from malt bagasse hydrolysate. Process optimization identified distinct nutritional requirements for each strain, while hydrolysate detoxification significantly improved biomass and lipid production, which was more evident during scale-up in a 1.0 L bioreactor. In addition to increasing lipid levels, detoxification altered the fatty acid profile, resulting in higher proportions of saturated fatty acids (C10:0, C16:0, and C18:0). This alteration in fatty acid profile may be advantageous for applications such as the production of oleochemicals and sustainable aviation fuels (SAFs).

Although not aimed at biotechnological applications, Chávez-Tinoco et al. (2024) developed a gene-editing platform for *C. maltosa*. Triple auxotrophic strains (with deletions of the HIS1, LEU2, and ARG4 genes) were generated using the SAT1-flipping strategy, previously optimized for *C. tropicalis*, representing a significant milestone for future metabolic engineering efforts. The successful sequential deletion of both EFG1 alleles not only confirmed the platform’s applicability but also revealed functional divergence compared to pathogenic *Candida* species, in which Efg1 promotes filamentation. In *C. maltosa*, conversely, the transcription factor represses pseudohyphae formation, demonstrating that regulatory circuits cannot be directly extrapolated from related species. This finding underscores the need for species-specific functional studies before rational engineering strategies can be developed.

Overall, these studies indicate that *C. maltosa* serves as a versatile platform for industrial biotechnology. Strategies such as ALE, cultivation optimization, and genetic editing provide a foundation for significant biotechnological applications, including the production of fuels, oleochemicals, biosurfactants, and other high-value-added compounds.

**Table 4:** – Summary of biotechnological application studies of *Candida* non-*albicans* focused on cultivation strategies

Species	Strain	Strategy	Product (Titer, Yield and/or Productivity)	Reference
*C. tropicalis*	DSM 7524	Batch and fed-batch cultivations on pure xylose medium	Xylitol (86.8% yield from xylose)	Tamburini et al. 2015
	NBRC 0618	Batch cultivations on olive tree pruning biomass hydrolysate	Ethanol (27 38% yield from glucose) Xylitol (12 to 23% yield from xylose)	Mateo et al. 2015
	X828	Batch cultivation on mixed culture with *Bacillus subtilis* in xylose mother liquor	Xylitol (95 g/L and 75% yield from xylose)	Wang et al. 2016
	NCIM 3123	Batch cultivation on corn cob hemicellulosic hydrolysate	Xylitol (41,0 g/L)	Yewale et al. 2016
	MTCC 25,057	Batch cultivation in consolidated bioprocessing (CBP) approach with wheat straw hydrolysate	Enzymes (114.1 U/g of exoglucanase, 97.8 U/g of endoglucanase, and 689.3 U/g of xylanase) Xylitol (15.8 g/L) Ethanol (7.3 g/L)	Mattam et al. 2016
	MTCC 6192	Batch cultivation on corn cob hydrolysate	Xylitol (85% yield)	Kumar et al. 2018
	31,949	Batch cultivation on sugarcane bagasse hydrolysate	Xylitol (62.98 g/L)	Xu et al. 2019
	JA2	Batch cultivation on sugarcane bagasse hydrolysate with high acetic acid content	Xylitol (86% yield, 109.5 g/L and 2.81 g/L h)	Morais Junior et al. 2019
	-	Batch cultivation on sugarcane bagasse hemicellulosic hydrolysate	Xylitol (74% yield, 21.1 g/L, and 0.24 g/L h) Arabitol (43% yield, 4.2 g/L, and 0.02 g/L h)	Beviaqua et al., 2023
	MTCC 6192	Batch cultivation on areca nutshell hydrolysate	Xylitol (5.88 g/L, 79.68% yield)	Vardhan et al. 2023
	FT120037	Batch cultivation on sugarcane bagasse/straw hydrolysate with yeast rapid adaptation	Xylitol (67% yield, 0.73 g/L h	Bianchini et al. 2023
	GS18	Batch cultivation on hemicellulosic and cellulosic fractions of *Arundo donax*	Xylitol (54% yield, 0.274 g/L h) Ethanol (47% yield, 0.63 g/L h)	Sidana et al. 2024
	FTI 20,037	Batch cultivation on defined medium (optimization of glucose/xylose ratio and pH)	Xylitol (29.81 g/L, 54% yield, 0.52 g/L h)	Queiroz et al. 2025
	ATCC 1369	Batch cultivation on rice husk hydrolysate with rapidly adapted yeast	Xylitol (58% yield, 36.74 g/L, 0.34 g/L h)	Eraso et al. 2025
	S7	Batch cultivation on banana scutcher hydrolysate	Xylitol (81% yield)	Kumar et al. 2026
	HNMA-1	High-temperature batch cultivation on xylose and sugarcane bagasse hydrolysate	Ethanol (7.6% yield from xylose and 7.8% yield from hydrolysate)	Nouri et al. 2017
	TISTR 5306	Batch and fed-batch cultivation on longan waste juice and extract	Ethanol (25.5 g/L on batch cultivations and 28.3 g/L on fed-batch)	Mahakuntha et al. 2021
	ATCC 20,962	Fed-batch cultivations with glucose and grease trap waste	Long-chain dicarboxylic acids (4.7 g/L, 0.42 g/L h)	Gilis et al. 2026
	ASY2	Batch cultivation on cassava processing wastewater	Lipids (49% w/w, 0.010 g/L h)	Thangavelu et al. 2020
	SY005	Batch cultivation on glucose and xylose defined media	Lipids (0.792 g/L d)	Chattopadhyay and Maiti 2020
*C. parapsilosis*	SK26.001	Fed-batch cultivation on glucose defined medium	Mannitol (97.1 g/L)	Meng et al. 2017
	27RL-4	Batch cultivation on L-arabinose or glucose defined medium	Arabitol (10.72 g/L; 53% yield)	Kordowska-Wiater et al. 2017
	-	Batch cultivation on contaminated dairy products	Biosurfactant (13-docosenamide)	Garg et al., 2018
	CMGB-YT	Batch cultivation on oil-polluted soil	Biosurfactant (Glycolipid)	Csutak et al. 2024
	NH-3	Batch cultivation on sugarcane molasses	Citric Acid (4.3 g/L; 9% yield)	El-Gamal et al. 2018
*C. viswanathii*	-	Batch cultivation on Amazon vegetable oils	Lipase (1.66 U/mL)	Gomes et al. 2018
	Y-E4	Fed-batch cultivation on glucose defined medium	Lipids (50% w/w)	Ayadi et al. 2016
	Y-E4	Batch cultivation on crude glycerol (biodiesel byproduct)	Lipids (52% w/w)	Guerfali et al. 2020
	IPE-1	Batch cultivation on wheat straw hydrolysate and n-dodecane	Dodecanedioic Acid (128.7 g/L and 1.13 g/L h)	Cao et al. 2017
	pFP19C	Fed-batch optimized cultivations on soy peptone, trace elements, vitamins and glycerol	Dodecanedioic Acid (90–162 g/L)	Pham et al. 2026a
*C. sojae*	JCM 1644	Two-stage fermentation of Brassica juncea (mustard) hydrolysate	Ethanol (45% yield) Xylitol (62% yield)	Pant et al. 2022
*C. maltosa*	NRRL Y-17,677	Batch cultivations on media containing polyethylene derived compounds	Single-cell protein	Brown et al. 2023
	UFV-1 e UFV-2	Batch cultivations in non-detoxified and detoxified malt bagasse hydrolysate	Lipids (18.2% w/w, 3.14 g/L titer, 0.05 g/L h for UFV-1; 12.2% w/w, 1.58 g/L titer, 0.03 g/L h for UFV-2)	Freitas and Dias et al. 2026
	NRRL Y-17,677	Batch cultivations in minimal media containing polyethylene derived compounds supplemented with algae extract, casamino acids or ammonium sulfate	Single-cell protein	Noorozi et al. 2026

**Table 5:** **–** Summary of metabolic engineering efforts in *Candida* non-*albicans* with biotechnological potential

Species and strain	Strategy	Target gene(s)	Product of interest	Reference
*Candida parapsilosis* DSM 70,125	Genome shuffling	Random	Arabitol	Kordowska-Wiater et al. 2018
*Candida tropicalis*	Adaptive laboratory evolution	Random	Xylitol	Li et al. 2015
*Candida tropicalis*	Adaptive laboratory evolution	Random	Xylitol	Kaur et al. 2023
*Candida tropicalis*	Adaptive laboratory evolution	Random	Lipids	Luo et al. 2023
*Candida tropicalis* 1798	Knockout vector	CRAT gene	Long-chain Dicarboxylic Acids	Wang et al. 2018
*Candida tropicalis* KCTC 7212	Overexpression	AOX4 AOX5	Adipic acid	Ju et al. 2020
*Candida tropicalis* SY005	Ectopically expressing	CtRAP1	Lipid	Chattopadhyay et al. 2020
*Candida tropicalis* 1798	Homologous recombination for knockout and overexpression	ctfat1p e ctpxa1p	Long-chain Dicarboxylic Acids (DCAs)	Zhang et al. 2021a, b
*Candida tropicalis* ATCC 20,336	Gene deletion and heterologous expression using a recombination system	XYL2 (knockout) YIZWF, YIGND and YIMAE (heterogous expression)	Xylitol	Zhang et al. 2021a, b
*Candida tropicalis* ATCC 20,336	CRISPR-Cas9	α-humuleno sintase (ZSS1) and mevalonate pathway genes	α-Humulene	Zhang et al. 2022
*Candida tropicalis* ATCC 20,336	CRISPR-Cas9	URA3 GFP3 ADE2 ERG9	β-Caroteno	Li et al., 2023
*Candida tropicalis* K2	EMS mutagenesis and Knockout	XYL2/xyl2Δ	Xylitol and ethanol	Singh et al. 2023
*Candida tropicalis* CU-208	CRISPR-Cas9	CBTS1	Cembratriene-ol (CBT-ol)	Zhang et al. 2024
*Candida tropicalis* ATCC 20,336	Homologous recombination	POX5 (Knockout), LS, ILS, ERG20, ERG20WW (Integration/Overexpression)	L-limonene	Yang et al. 2024
*Candida tropicalis* BT06	Overexpression vector	XylD	1,2,4-butanetriol	Li et al. 2024
*Candida tropicalis*	Scaffold-Mediated Assembly Regulation	Co-expression of MrBBS and ERG20 Regulation of eight acetyl-CoA/mevalonate pathway genes Knockout of DPP1, Cat2, ERG10, ADH1, GPD1.	(−)-α-bisabolol	Yang et al. 2026
*Candida viswanathii* ATCC 20,962	CRISPR	Ade2, POX2, CYP52A13/14, CYP52A15/16 (knockout) CYP52A18, CYP52A19, CPRb, FAO2, POS5, G6PD, 6-PGDH, GDP1, PNTA/PNTB (over/expression)	Dodecanedioic Acid (DDA), DA (Ácido Dodecenoico), HDA (Ácido Hidroxidodecanoico)	Pham et al. 2023
*Candida viswanathii*	dCasMINI-Med2	STL1_4 and HST6.	Dodecanedioic Acid (DDA)	Pham et al. 2026b
*Candida maltosa* Xu316	SAT1-flipping	EFG1	Filamentation and biofilm formation	Chávez-Tinoco et al. 2024
*Candida maltosa*	Adaptive laboratory Evolution	Random	Xylitol	Jiang et al. 2016
*Candida maltosa*	Adaptive laboratory Evolution	Random	Biosurfactant	Rodriguez-Ocasio et al. 2026

## Challenges and perspectives

There has been growing interest in non-*albicans Candida* species. For instance, *C. tropicalis* has been the most studied. This interest is due to their metabolic versatility, including the ability to assimilate hexoses, pentoses, glycerol, aromatic compounds, and hydrocarbons derived from agroindustrial byproducts, combined with high inhibitor tolerance. As such, non-*albicans Candida* species demonstrate great potential to be applied in bioprocesses aligned with principles of circular bioeconomy. Nevertheless, some challenges need to be circumvented to widely apply them as biotechnological platforms. First, although several strains are not clinically related and potentially suitable for bioprocesses after appropriate safety assessment, the phylogenetic proximity with pathogenic species demands careful strain selection and characterization. These concerns have also contributed to a slower expansion of research on non-*albicans Candida* species compared to other non-conventional yeasts, such as *Y. lipolytica*, *K. marxianus* and *R. toruloides*. Another major challenge regards the lack of deep understanding of the central and secondary metabolism of these yeasts. For most species, like *C. sojae*, *C. viswanathii*, and *C. maltosa*, functional information on regulatory pathways, transport systems, and stress-response pathways remain poorly characterized. Even in *C. tropicalis* and *C. parapsilosis*, which have been more studied, multi-omics investigations and mechanistic studies are scarce. These knowledge gaps impair strain design and the development of metabolic engineering tools.

Systems biology can contribute to reducing these knowledge gaps. The development of efficient genetic engineering platforms, such as CRISPR-Cas9, associated with omics and modeling approaches for non-model yeasts is crucial to developing novel strains. Nevertheless, the knowledge regarding the metabolism and genetics of non-*albicans Candida* species remains scarce, which impairs or difficult these efforts. The Design–Build–Test–Learn (DBTL) framework represents one of the most promising perspectives to gain metabolic and genetics insights while developing novel strains. High-throughput genome editing, automated phenotyping, and ALE, for example, can accelerate strain optimization and knowledge gains. Through DBTL cycles it will be possible to expand the application of non-*albicans Candida* species beyond xylitol, DCAs, lipids, and alcohols, into emerging sectors such as biopolymers, terpenoids, aromatic derivatives, advanced biofuels, and specialty chemicals.

## Data Availability

No datasets were generated or analysed during the current study.
